# Bringing rest into consideration: analyzing a database of computational models for multistability of oscillatory and stationary regimes

**DOI:** 10.1186/1471-2202-12-S1-P52

**Published:** 2011-07-18

**Authors:** Bóris Marin, William Barnett, Anca Doloc-Mihu, Ronald L Calabrese, Gennady Cymbalyuk

**Affiliations:** 1 Instituto de Física, Universidade de São Paulo, São Paulo, SP Brazil; 2 Neuroscience Institute, Georgia State University, Atlanta, GA 30303, USA; 3 Biology Department, Emory University, Atlanta, GA 30322, USA

## 

Biophysically realistic neuronal models are defined in high-dimensional parameter spaces. Brute-force database approaches have proven themselves fruitful in characterizing neuronal models [1,3,5]. In a brute-force database approach, the corresponding activities and parameters of the simulated neuronal models are stored in a database, which is further efficiently searched for insightful information about the models. We present a novel technique which combines a brute-force approach with bifurcation continuation methods from the theory of dynamical systems. We considered a database of single compartment neuron model that can display, silence, tonic spiking and bursting activity [3]. A one-parameter bifurcation analysis allowed us to determine stable and unstable stationary states for each case from the database. Using this novel database technique, we systematically tested models for the coexistence of stable stationary states and bursting activity.

Specifically we used this technique to extend the database describing the model of the isolated leech heart interneuron to include stationary states [3]. We took into consideration only those cases that were shown to produce functional half-center oscillators (HCOs) [2-4]. By using the leak conductance (g_leak_) as the controlling parameter for bifurcation analysis, we showed that the model could exhibit two stable depolarized stationary states and two hyperpolarized stationary states in addition to various spiking and bursting regimes. For different initial conditions as many as three different attracting regimes could be exhibited by a case of the model. We found cases demonstrating either bursting activity, hyperpolarized stationary state or depolarized stationary state.

We investigated the 2,387 cases that produced regular bursting activity, and found that 421 (18%) were multistable. We found that among the latter there were 361 cases which exhibited bistability of bursting and a hyperpolarized stationary state and 47 cases which showed bistability of bursting and a depolarized stationary state. Out of those 361 cases, one case showed bistability of bursting and a second, slightly less hyperpolarized stationary state. Twelve cases demonstrated tristability of coexisting bursting and two stable stationary states, hyperpolarized and depolarized.

Our method also allowed us to determine that 2,128 out of 2,387 (89%) cases are multistable for some range of *g_leak_* values. Among these cases, the majority, 2,078 (98%), showed at least one range of *g_leak_* values with two attracting regimes, bursting and a hyperpolarized stationary state.

We also explored the activity of HCOs built of multistable regular bursting model neurons (421 described above) in response to perturbations of the state variables. For a given set of initial conditions, 353 (84%) cases showed functional activity. We further explored the activity of these 353 cases. After 100 seconds of functional activity the state of one of the neurons was reset to be close to a stable stationary state, and the synaptic conductances determining the mutual inhibition of the HCO were set to zero for 5 seconds. This interruption of the normal HCO dynamics produced a perturbation of the state variables. In 96% of these cases, the HCOs exhibited functional bursting, proving itself robust against this perturbation too. Multistability of oscillatory regimes and stationary states poses a threat to functionality of central pattern generators which are required to produce a persistent pattern. The half-center oscillator configuration could serve as network-based protective mechanism against malfunctional multistability. On the other hand multistability of the dynamics of neurons could be functional providing a substrate for memory units, toggle switches, and elements of multifunctional central pattern generators.

BM is supported by the Brazilian agency FAPESP. GC and WB are supported by NSF grant PHY-0750456. AD-M and RLC are supported by NIH grant NS024072
